# A new apparatus to induce lysis of planktonic microbial cells by shock compression, cavitation and spray

**DOI:** 10.1098/rsos.160939

**Published:** 2017-03-15

**Authors:** A. Schiffer, M. N. Gardner, R. H. Lynn, V. L. Tagarielli

**Affiliations:** 1 Department of Mechanical Engineering, Khalifa University, Abu Dhabi, UAE; 2 Department of Earth Sciences, University of Oxford, South Parks Road, OxfordOX1 3AN, UK; 3 Department of Engineering Science, University of Oxford, Parks Road, Oxford OX1 3PJ, UK; 4 Department of Aeronautics, Imperial College London, London SW7 2AZ, UK

**Keywords:** shock wave, cavitation, *Escherichia coli*, cell lysis

## Abstract

Experiments were conducted on an aqueous growth medium containing cultures of *Escherichia coli* (*E. coli*) XL1-Blue, to investigate, in a single experiment, the effect of two types of dynamic mechanical loading on cellular integrity. A bespoke shock tube was used to subject separate portions of a planktonic bacterial culture to two different loading sequences: (i) shock compression followed by cavitation, and (ii) shock compression followed by spray. The apparatus allows the generation of an adjustable loading shock wave of magnitude up to 300 MPa in a sterile laboratory environment. Cultures of *E. coli* were tested with this apparatus and the spread-plate technique was used to measure the survivability after mechanical loading. The loading sequence (ii) gave higher mortality than (i), suggesting that the bacteria are more vulnerable to shear deformation and cavitation than to hydrostatic compression. We present the results of preliminary experiments and suggestions for further experimental work; we discuss the potential applications of this technique to sterilize large volumes of fluid samples.

## 1. Introduction

*Escherichia coli,* and bacteria in general, can represent a serious problem for many industrial processes; they can adversely affect process performance and pose a risk to public health by altering the local environment in which they exist [1]. The oil and gas industry, for instance, suffers from blockages in pipelines and filters caused by resilient surface-associated microbial communities forming biofilms that proliferate in organic-rich environments [2]. Similarly, the food industry encounters microbial contamination issues and has long resorted to cell disruption technologies for sterilization and purification purposes [3].

Heat sterilization to counter microbial contamination is not a viable option in all situations, particularly when it can compromise liquid products. When this is not a viable option, commonly used devices for mechanical cell disruption are based on the principle of the French pressure cell [4]. In its original form, this apparatus consists of a piston which slowly compresses a liquid sample containing microbial cells to a pressure of approximately 140 MPa, followed by ejection of the fluid through a needle valve at a flow rate in the range 2–16 ml min^−1^. Ejection subjects microbial cells to decompression and fluid shear, which causes their membranes to tear, resulting in cell lysis and death; however, the French press cannot be used to process large amounts of liquid.

An alternative to the French press was proposed by Hughes [5], who used an ultrasonic probe to generate pressure waves in a fluid sample. Sonic disintegration as developed by Hughes involves cyclic growth and collapse of cavitation bubbles at frequencies in the range 20–50 kHz. Hughes used yeast as the test organism and found significant cell disruption in his samples. He noted that the effectiveness of the method increased when small glass particles and air bubbles are added to the sample, consistent with the notion of lysis being induced by cavitation.

Several researchers have attempted lysing living cells using pressure shock waves; the first studies on this subject date back to the ‘hammer, cork and bottle’ experiment of Ahlborn *et al.* in 1895 [6]. Recently, Loske *et al*. [7] used an electrohydraulic shockwave generator to subject water samples containing *E. coli* to repetitive shock loading. The survivability of bacteria was determined by spread-plate analysis, with a significant reduction in living bacteria population reported. From this study the authors suggested cell disintegration may be driven by the rise and collapse of cavitation bubbles.

Some researchers [7–10] have conducted impact tests on bacteria to test the hypothesis of panspermia, i.e. that living organisms could have been transported to Earth by meteorites or asteroids. For this to be possible, microorganisms would need to resist the enormous pressures experienced during planetary impact. Early work in this field was conducted by Burchell *et al*. [8], who conducted hypervelocity impact experiments to replicate the high pressures experienced by microorganisms during meteorite impact. It was shown that soil bacteria (*Rhodococcus erythropolis*) can be accelerated to the speed of 5 km s^−1^ (approximately the escape velocity of Planet Mars) and survive subsequent impact into agar with survival rates on the order of 10^−7^. Horneck *et al*. [9] used an explosive laboratory set-up to subject bacterial spores (*Bacillus subtilis*) to peak pressures up to 32 GPa, reporting survival rates in the range 10^−6^ to 10^−7^. Later, Burchell *et al*. [10] successfully recovered viable microorganisms (*R. erythropolis* and *B. subtilis*) from porous ceramic samples after firing them at targets of agar and ice at speeds of 5.4 kms^−1^, inducing shock pressures of 78 GPa upon impact. They found that the *B. subtilis* (survival rate 1.8 × 10^−5^) were more resilient to shock compression than the *R. erythropolis* (survival rate 8.8 × 10^−8^). Investigating species-specific responses, Stöffler *et al*. [11] subjected samples of bacterial spores (*B. subtilis*), cyanobacteria (*Chroococcidiopsis* sp.) and lichens (*Xanthoria elegans*), sandwiched between thin layers of rock, to shock pressures between 5 and 50 GPa, in order to mimic a meteorite impact. Finite survival rates on the order of 10^−7^ were reported for *B. subtilis* and *X. elegans* at peak pressures of 50 GPa, whereas the *Chroococcidiopsis* cells did not survive pressures above 10 GPa.

For liquid medium investigations, Willis *et al*. [12] conducted flyer plate experiments on live colonies of 10^7^ *E. coli* suspended in 10 mm^3^ water-based growth media, impacting the samples at velocities in the range of 0.6–1.5 km s^−1^, and inducing peak pressures of up to 15 GPa. While no survivability was reported for shock pressures of 15 GPa, a significant proportion of viable cells (10^−2^ to 10^−4^) survived peak shock pressures of 220 to 260 MPa applied for a duration of 800 ns. Tunnelling electron microscope (TEM) images of shock recovered bacteria suggested that rupture and delamination of the cell walls may have been the main mechanism disrupting cellular integrity.

Recently, Price *et al*. [13] investigated the survivability of yeast in spore form (*Saccharomyces cerevisiae*) to hypervelocity impact by firing BY4743 spores into targets of water at velocities ranging from 1 to 7.4 km s^−1^, corresponding to peak shock pressures of approximately 43 GPa. It was shown that by exposing the samples to peak pressures of 1.2 GPa for approximately 1 µs, the surviving fraction of spores was on the order of 10^−1^, while it dropped to 10^−3^ after applying a peak pressure of 24.8 GPa for 0.3 µs on the samples. Based on their findings and the results of previous studies, they proposed an empirical model able to predict the effect of peak pressure on the survivability of various types of species. They also noted that the survivability of microorganisms follows two regimes (in line with the findings of previous studies, see for example Burchell *et al*. [14]): (i) a regime at low shock pressures where the survivability decreases modestly and linearly with increasing peak pressure, and (ii) a regime where the survivability decreases logarithmically at higher shock pressures.

In the above liquid medium experiments, the microbial cultures were subjected to different sequences of high-pressure shock loading, water cavitation, shear deformation and temperature rise; this represents a problem as it makes unclear to what extent each mechanism is responsible for the loss of cellular integrity. To control cavitation, Hazell *et al.* [15] designed a metal capsule which could be used as a target in plate-impact experiments. These experiments would allow for liquid bacterial suspensions to be subjected to shock pressures of up to 1.2 GPa, with cavitation optionally suppressed or allowed. The authors performed numerical simulations to estimate the pressure and temperature history in the target chamber, as well as the possible occurrence of water cavitation. When using *E. coli* (NCTC 9001), *Enterococcus faecalis* (ATCC 19433) and *Zygosaccharomyces bailii* (DSM 70492), it was found that with cavitation fully supressed these microorganisms were not affected by shock compression, suggesting that water cavitation might have the greater potential to disrupt the cells. Subsequently, similar experimental techniques [15] were used to study the shock response of different biological systems [16–18].

Many of the experimental techniques outlined have limitations in sample volume and do not allow control of the exact pressure histories imposed on the bacteria; these need to be deduced from numerical simulations or simplified theoretical calculations. In addition, considerable heat is generated in high-velocity impact experiments. For these reasons, the results available in the literature do not allow for a clear assessment of the extent by which different loading mechanisms (i.e. shock compression, cavitation, shear flow, temperature rise, etc.) contribute to cell lysis.

In this paper, we employ a new bespoke shock-tube apparatus; this has been previously developed to explore the dynamic response of different types of structures to loading from underwater shock waves owing to explosions [19–22], but is used here in a modified version to measure the survivability of bacteria following two distinct loading sequences: (i) shock compression followed by cavitation, and (ii) shock compression followed by spray. In one single test, different portions of a liquid samples are subjected to the two sequences above. We present results from preliminary experiments conducted with this new apparatus; we discuss the implications of the results on the resilience of bacteria to different types of mechanical straining; finally, we propose further work and comment on potential industrial applications of the presented technique.

## 2. Experimental techniques

### 2.1. Test organisms

The rod-shaped bacterium used in this study is the Gram-negative *E. coli* XL1-Blue (Stratagene), which has a typical width of 0.5 µm and length of 2 µm [1].

It is known that bacterial cell membranes undergo various morphological changes during batch culture growth resulting in different degrees of resilience to mechanical loading and environmental stress [23]. Consequently, it is necessary to monitor the growth stage of the bacteria tested. For this reason, we employed the standard microbiological technique of measuring culture turbidity at 600 nm using a spectrophotometer. The planktonic culture turbidity is proportional to bacterial biomass and indirectly proportional to cell number. Being able to plot turbidity versus culture incubation time allows one to identify the different phases of culture growth, namely: (i) lag phase, (ii) exponential growth phase, (iii) stationary growth phase, and (iv) a death phase [1].

All shock loading experiments and their respective loading sequences were undertaken in duplicate, using the laboratory model bacterium *E. coli* XL1-Blue cultured in flasks of tryptic soy broth (acquired from Cole-Palmer UK and containing peptone from casein 15 g l^−1^, peptone from soymeal 5 g l^−1^, sodium chloride 5 g l^−1^, agar-agar 15 g l^−1^) and incubated at 37°C in an orbital shaker at 120 r.p.m. The measured average density of the soy broth was only marginally larger than that of water, *ρ_w _*= 1030 kg m^−3^.

### 2.2. Mechanical loading apparatus

**Figure 1.**
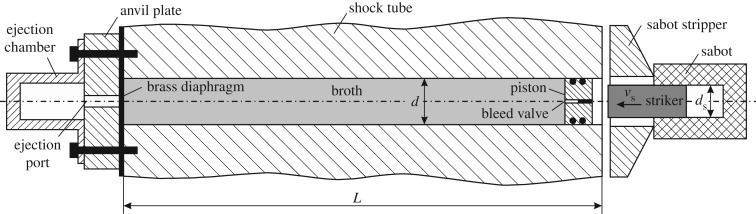
Schematic of the experimental apparatus used to subject *E. coli* to mechanical loading.

In this study, a bespoke shock tube was developed to subject the bacterial cultures to shock compression followed by either fluid cavitation or ejection through a small hole (to which we refer, in this paper, as ‘spray’). The apparatus consisted of a stainless steel shock tube machined to a length of *L *= 1 m, outer diameter *D *= 150 mm and bore diameter *d *= 10 mm, as sketched in figure 1. It was closed at one end by a thin brass diaphragm of thickness *h *= 25.4 µm, clamped to the annular end of the tube by a bolted steel cap. The cap was machined with a central hole of diameter *d*_c_* *= 4 mm and length 10 mm to allow jet ejection of the liquid samples upon bursting of the brass diaphragm. To capture the ejected portion of the test sample, a circular cylindrical hollow chamber was machined from stainless steel and bolted onto the steel cap; a valve was included on the top face to vent air during the spray process.

The tube was then filled in a vertical position with a broth containing the bacterial culture, and closed at the opposite end by a sterilized sealing steel piston of mass *M*_P_ = 2.1 g. The piston was fitted with two O-rings and included a bleed valve to allow evacuation of air bubbles trapped in the tube prior to the dynamic experiment (this is necessary to avoid attenuation of the shock waves generated, [24]).

Exponentially decaying pressure pulses were generated in the broth by firing a steel projectile of mass *M*_s_ = 7.2 g and velocity *v*_s_ = 236–282 ms^−1^ at the dry face of the steel piston using a single-stage gas gun. The projectile was carried by a nylon sabot which maintained an air-tight seal with the gun barrel. A steel device located at the muzzle end stripped the sabot from the projectile, which briefly continued in free flight before impacting the piston, thereby sending an exponentially decaying compressive shock wave through the test sample.

It can be shown mathematically [24] that this shock wave decays exponentially as

2.1 $$p(t)=p_{0}\exp\left(\frac{-t}{\theta }\right),$$

with peak pressure and decay time given by

2.2 $$p_{0}=\rho_{w}c_{w}v_{0},\;\theta =\frac{m_{S,P}}{\rho_{w}c_{w}},$$

respectively, where *m_S_*_,*P*_ = (*M*_S_ + *M*_P_)/*A*_P_ is the combined areal mass of the striker-piston system, related to the cross-sectional area of the shock tube *A*_p_, and *v*_0_ is the initial system velocity governed by conservation of linear momentum, *v*_0 _= *M*_S_*v*_S_/(*M*_S _+ *M*_P_). The density and speed of sound in the liquid test sample are *ρ_w_* and *c_w_*, respectively. The decay time *θ* and the peak pressure *p*_0_ can be independently adjusted by adjusting mass and velocity (respectively) of the projectile, allowing generation of a controllable shock, see [20,21,24].

### 2.3. Shock wave measurements

Preliminary experiments were conducted with the apparatus to check the pressure profiles generated. In these experiments, the ejection chamber and the brass diaphragm were not used, and the steel cap was replaced by a calibration plate machined from stainless steel and bolted onto the distal face of the tube. A central mounting port was included to mount a high frequency pressure gauge (PCB Piezotronics, model 113B03, 15 k psi range, more than 500 kHz resonant frequency) flush to the inner surface, allowing for dynamic pressure measurements at this end. Steel projectiles of mass *M_S _*= 7.2 g were accelerated in the gas gun to impact the target at velocities between 80 and 270 ms^−1^, generating exponentially decaying shock waves according to equation (2.1). If one ignores the small deformation of the thick steel end-plate, conservation of linear momentum dictates that when such a wave reaches the fixed end of the tube, the pressure applied on the fluid–structure interface is

2.3 $$p_{f}(t)=2p_{0}\exp\left(\frac{-t}{\theta }\right).$$

### 2.4. Speed of shock wave propagation

To measure the speed of pressure wave propagation in the aqueous growth medium, we conducted a preliminary experiment using a smaller acrylic shock tube (outer diameter *D *= 70 mm, bore *d *= 27 mm, as described in [19]) filled with tryptic soy broth which was not inoculated with the bacterial culture (to avoid contamination of the laboratory facility). In this experiment, we employed the same methodology as described above; however, owing to sanitary requirements, we used a sliding piston at the end of the tube (instead of the diaphragm and ejection chamber) which maintained a tight seal with the tube wall (as in [19]).

A shock wave (equation (2.1)) was generated by firing a steel striker at the front piston and the pressure transient in the tube was measured at two locations using piezoelectric pressure gauges (PCB Piezotronics, model 113B23). We found that the speed of shock wave propagation was almost identical to that of filtered water (see [19]), suggesting that the magnitude and decay of shock waves (equation (2.2)) generated by striker impact are similar in both media. It is important to note that because the measured speed of shock wave propagation in the compliant acrylic tube is expected to be lower than in the stiff steel tube (see [19]), we assume *c*_w_ = 1400 ms^−1^ for the bacterial broth in the following calculations, in line with similar experimental studies [24].

### 2.5. Observation of cavitation processes

Previous cell disruption studies suggest the occurrence of cavitation processes may contribute to the lysis of bacteria in liquid suspensions, as discussed in §1. To visualize the cavitation processes active in the experiments, we conducted additional tests using a transparent shock tube (outer diameter *D *= 70 mm, bore *d *= 27 mm, see [19]), which allows observing cavitation processes in the liquid by means of high-speed photography. In these experiments we employed the same methodology described above, with the steel cap and ejection chamber replaced by a transparent ring of inner diameter *d *= 20 mm clamped to the distal face of the shock tube to hold in place a brass diaphragm.

A high-speed camera (Vision Research v. 7.1) was employed to record the response of the fluid subsequent to bursting of the diaphragm (figure 3). Owing to sterility requirements, this test was carried out with filtered water rather than bacterial broth; however, speed of sound and density of water are similar to those of the bacterial broth (see §2.4) and therefore the fluid–structure interaction processes observed in these tests are representative of those active in the case of studies using bacterial broth.

### 2.6. Shock-test protocol

Prior to shock testing, all parts of the apparatus in figure 1 were sterilized *in situ* with absolute ethanol of molecular biology grade. For each test performed, 200 ml of sterile tryptic soy broth was inoculated with 100 µl of an *E.coli* starter culture, and incubated at 37°C in an orbital shaker (120 r.p.m.) until the desired growth phase was reached. The turbidity was measured at different times by spectrophotometry at 600 nm, the sterilized shock tube was filled with the bacterial culture and closed at one end with the system of diaphragm, steel cap and ejection chamber (figure 1); the opposite end was sealed with a sterile steel piston. The remainder of the bacterial culture was kept at room temperature to provide a reference sample for survivability measurements. Immediately following the mechanical loading test, samples of the bacterial culture were taken from both the ejected and non-ejected fluid, and the percentage survivability determined by viable cell counts, as detailed below.

### 2.7. Viable cell count measurements

The standard microbial spread-plate technique [12,25] allows for calculation of the number of viable bacteria in a broth. To do so, one assumes that each colony that grows on an agar plate originates from a single bacterial cell. In our studies, 100 µl of our diluted test samples were plated onto a tryptic soy agar plate and incubated overnight at 37°C. Following the incubation, we counted the number of visible bacterial colonies on the agar plate and expressed the total viable cell count as colony forming units per ml of broth (CFU ml^−1^). By comparing the CFU counts of the ejected, non-ejected and reference samples, the percentage survivability for each ejected and non-ejected sample was calculated as follows:

2.4 $$s_{\mathrm{ejected}}=\frac{{\text{CFU}}_{\mathrm{ejected}}}{{\text{CFU}}_{\mathrm{reference}}}\;\text{and}\;s_{\mathrm{non}\text{-}\mathrm{ejected}}=\frac{\text{CF}{\text{U}}_{\mathrm{non}\text{-}\mathrm{ejected}}}{\text{CF}{\text{U}}_{\mathrm{reference}}}.$$

## 3. Results

**Figure 2.**
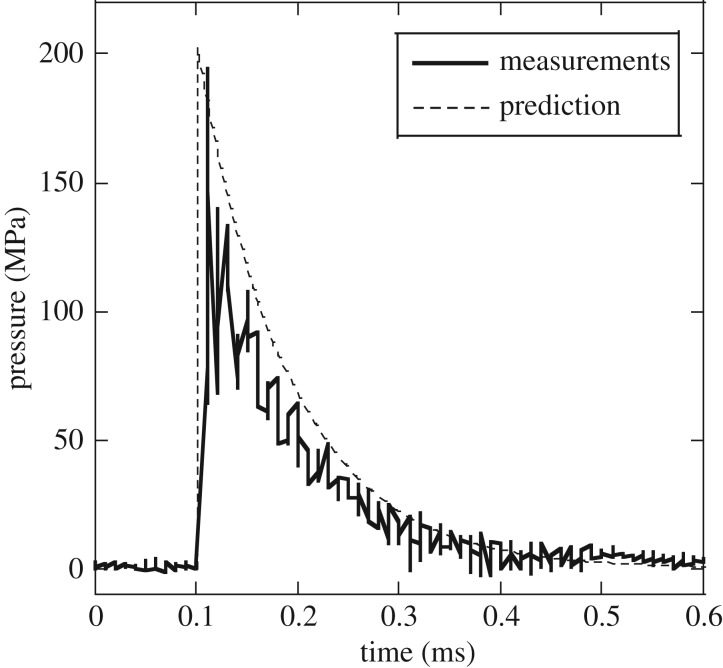
Pressure versus time as measured at the capped end of the tube during the calibration experiment; theoretical predictions are included for comparison.

In figure 2, we present pressure versus time histories directly measured, as described in §2.3, in an experiment with *v*_0 _= 82 ms^−1^; figure 2 includes the corresponding theoretical predictions (equation (2.3)). The predictions are for the choice *ρ_w _*= 1030 kg m^−3^ and *c_w _*= 1400 ms^−1^ for density and sonic speed of the bacterial broth, respectively. The measurements in figure 2 show a sudden rise in pressure to a peak value of *p*_0 _= 195 MPa, followed by an exponential decay. The predictions are in good agreement with the measurements, confirming that the design of the shock tube apparatus is adequate to perform liquid shock experiments in a controllable manner.

We note that the shock wave has a finite initial rise time of 10 µs. Because the speed of sound is *c_w_* = 1400 ms^−1^, we can conclude that as the wave advances in the fluid column there is a spatial region of length 14 mm over which the pressure rises from atmospheric to its peak value. This length is substantially greater than the average length of the bacteria, such that the maximum spatial variation of pressure over the volume occupied by a bacterium is small, of order 1 bar in these experiments and hence much smaller than the peak pressure. Therefore, it can be assumed that each bacterium in a planktonic culture experiences a spatially uniform rise in pressure across the volume it occupies.

**Figure 3.**
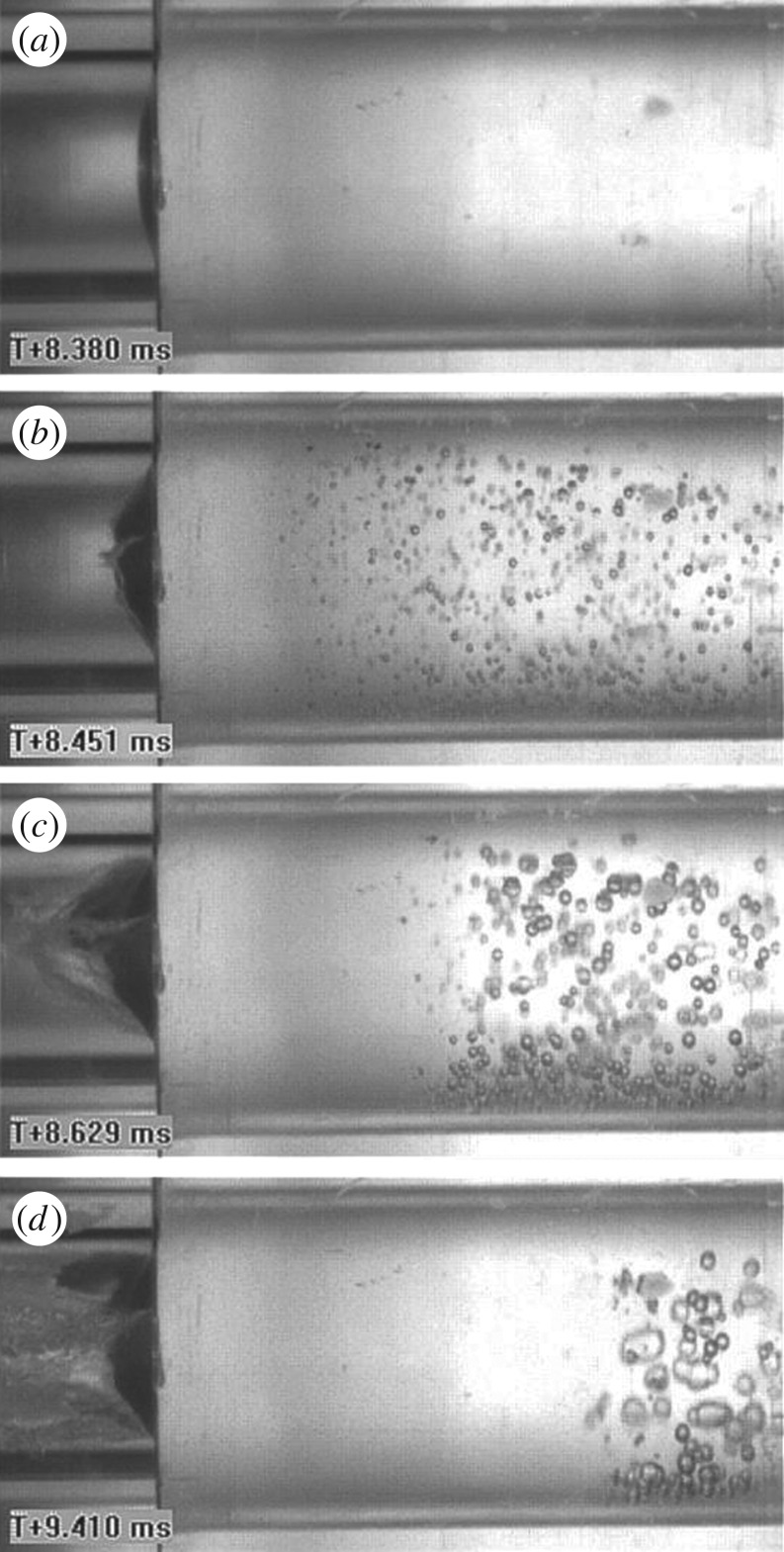
Sequence of high-speed photographs recorded with a transparent water shock tube shows evidence of cavitation in the water upon bursting of a thin brass diaphragm. The number shown in each frame indicates the time elapsed from triggering of the high-speed camera. (*a*) *T *= 8.380 ms; deformation of the diaphragm begins. (*b*) *T *= 8.451 ms; onset of failure, emergence and expansion of a cavitation zone. (*c*) *T* = 8.629 ms; ejection of water and collapse of the cavitated region. (*d*) *T *= 9.410 ms; further collapse of the cavitation zone.

Figure 3 presents results of a preliminary experiment to visualize the cavitation processes, performed as described in §2.4. Figure 3*a* shows the brass diaphragm and the fluid column during the initial phase of the response, shortly after the shock wave has impinged on the fluid–structure interface. No evidence of cavitation is observed within this phase of response, and the diaphragm has slightly deformed. In figure 3*b*, rupture of the diaphragm is first observed, leading to rapid acceleration of the adjacent fluid particles. Consequently, a tensile rarefaction wave [26,27] of considerable magnitude is sent into the fluid, causing the fluid pressure to drop below the water vapour pressure and giving rise to cavitation at a finite distance from the fluid–structure interface. The cavitation region spreads quickly to the entire volume of fluid in the tube and subsequently collapses, as is shown in figure 3*b*–*d*.

The process of expansion and collapse of a cavitation zone visible in figure 3 is strongly coupled with the motion of the diaphragm, as discussed extensively in [26,27]. A similar process is expected in the case of bacterial broth subject to high-velocity ejection from the steel shock tube. In these experiments, part of the broth is ejected through the small hole and part remains in the steel tube. The ejected fluid undergoes an exponentially decaying compressive shock, owing to arrival of the shock wave, followed by decompression and high shear deformation, associated with the ejection. Conversely, the non-ejected fluid undergoes the same compressive shock, followed by a process of expansion and collapse of a region of fluid cavitation. We proceed to explore the effect of these two distinct loading sequences upon cell integrity.

We present in table 1 the CFU counts for six different shock experiments. All tests were conducted at the highest impact velocity deemed safe for the equipment; the impact velocity was in the range 230–278 ms^−1^, giving calculated peak shock pressures in the range 263–310 MPa. Note that the data reported in table 1 correspond to three duplicated experiments conducted on bacteria at three different growth stages (corresponding to the three values of incubation time provided).

**Table 1.** Summary of shock experiments conducted.

			colony forming (10^7^ units ml^−1^)		
test	incubation time (h)	growth phase	reference	non-ejected	ejected
1	6	late exponential	31	20	17
2	6		25	24	11
3	6.5	late exponential/stationary	38	14	11
4	6.5		32	20	7
5	7.5	late stationary	53	29	3
6	7.5		57	21	7

**Figure 4.**
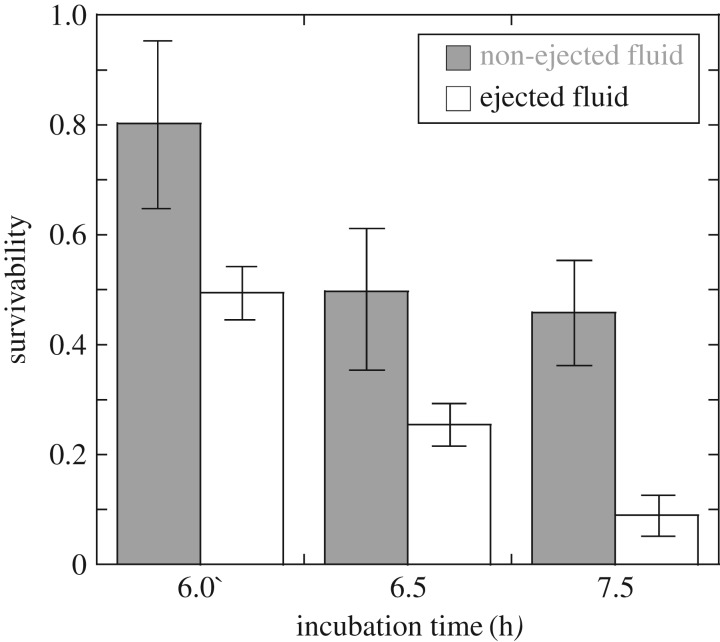
Average and scatter of the measured survivability of the bacteria after different incubation times, for both ejected and non-ejected samples.

The CFU counts determined before and after shock loading support the notion that mortality is induced by the shock loading, and that the direct measurement of the effects of the two loading sequences is possible. In figure 4, we report the measured survivability of the bacteria at different incubation times, for both ejected and non-ejected fluid. While the scatter in the replicated measurements is relatively high, and only two repeated tests were performed at different incubation times, the ejected bacteria present a lower survivability than non-ejected ones; this suggests that the action of shock compression followed by spray is more effective than shock compression followed by cavitation.

The average survivability at 6, 6.5 and 7.5 h of incubation was of 0.79, 0.49, 0.45 for the non-ejected samples and of 0.50, 0.25, 0.09 for the ejected samples, respectively. Analysis of this limited preliminary data suggests that survivability may be affected by the physiological state of the cell; it is well known [1] that *E.coli* undergo several morphological changes in the cell membrane as they proceed through different growth stages. The data reported in this study suggests a higher vulnerability of the cells following longer incubation times; such data are however very limited, and a scientific investigation of this aspect would necessitate a more extensive experimental campaign, which is left as a topic for future studies.

## 4. Discussion

In this section, we discuss the details of the mechanical loading experienced by bacteria in the ejected and non-ejected fluid and the potential application of this technique.

### 4.1. Non-ejected fluid

Bacteria in the non-ejected fluid experience a sequence of shock compression followed by growth and collapse of a cavitation region. In the first phase, they are loaded by a spatially uniform pressure of order 300 MPa increasing rapidly (over approx. 10 µs) and then decaying exponentially to atmospheric pressure relatively slowly (approx. 500 µs). As the bulk modulus of water is of order 2 GPa, the peak pressure applied in the experiments corresponds to a peak hydrostatic compressive strain of approximately 15%. This is probably not sufficient to disrupt the cellular membrane, as the results of previous studies suggest [15]. In the second phase, the liquid cavitates causing nucleation and rapid growth of gas bubbles (figure 3); subsequently, the cavitation is suppressed and the bubbles collapse. The process of expansion and collapse of cavitation bubbles occurs over a time period of milliseconds. During the cavitation process, the fluid first experiences a peak tensile pressure of approximately one atmosphere, as the pressure drops from atmospheric to the water vapour pressure, corresponding to a negligible tensile volumetric strain; subsequently, it undergoes a re-compression phase of magnitude less than 300 MPa. In the vicinity of the bubbles however, the bacteria also experience transient shear strains over a time period of milliseconds. It is known that the fast collapse of a cavitation bubble can produce high temperatures locally [7], however these occur only at the centre of the collapsing bubble (within a region of size 1 µm or less, and for very limited time, of order nanosecond) and are therefore unlikely to substantially heat the bulk volume of the test sample. Measurements on the broth prior and subsequent to the tests showed in fact a change in temperature of less than 5 K. It can be conjectured that only bacteria in the proximity of the tube wall, where cavitation bubbles are very likely to nucleate, grow and collapse (owing to the existence of microscopic bubbles at the solid boundary [28]), undergo lysis owing to the high shear strains and strain rates experienced. This hypothesis would need to be tested by conducting experiments on tubes of different bore diameters.

### 4.2. Ejected fluid

The bacteria in the ejected liquid initially experience the shock compression loading phase described above; subsequently, they accelerate to high speeds and experience high shear strains for a time period on the order of a millisecond, while they leave the shock tube and are sprayed through the hole. Once in the ejection chamber, the fluid jet undergoes high turbulence, again inducing further shear strains on the bacteria. The magnitude of the shear strains experienced in this phase is greater than that experienced by the non-ejected fluid.

Despite the controllable experiments conducted here, the strain history on the bacteria remains complex and difficult to quantify. The considerations above and the results of our preliminary study suggest that bacteria are less vulnerable to the hydrostatic strains induced by shock compression and more sensitive to the shear strain induced by both cavitation and spray, and this is reflected in the measured percentage survivability.

Our apparatus offers significant advantages over existing cell disruption techniques for the study of mechanical loading in liquid media: (i) it allows direct measurements of the applied loading profile and exploring its effect on cell integrity; (ii) organisms are contained within a sterile and practically isothermal shock tube during the experiment, thereby minimizing the risk of sample contamination and allowing for further studies after mechanical loading; (iii) the loading induces negligible heating of the bacteria; and (iv) its reliance on the propagation of an elastic wave, sweeping the sample at the speed of sound, makes the technique adequate for a cost-effective treatment of large volumes of contaminated liquid, often required in industrial applications.

### 4.3. Industrial application of the technique

From the point of view of the industrial applications of the technique presented, the limited tests presented in this study show that cell disruption by compressive shock followed by cavitation (non-ejected fluid) induces an average survivability of 57% (*s* = 0.57), whereas the compressive shock followed by spray (ejected fluid) is associated with an average survivability of 28% (*s* = 0.28). While the latter loading sequence gives smaller survivability, it is only applicable to treating the very small volumes of ejected fluid; the ejected fluid represents a fraction of 1–5% of the total volume of the test sample, of 78 cm^3^; we also note that the ejected fraction, of order 1 cm^3^, would not increase but rather remain constant, if the total volume of the sample were increased.

By contrast, the sequence of shock compression followed by cavitation is imposed to the entire volume of non-ejected fluid; using a longer tube and therefore larger fluid sample would not change this, and all fluid remaining in the tube would experience the same loading. This is because the compressive shock wave moves at sonic speed through the fluid with a very small attenuation, subjecting the whole liquid sample in the tube to high pressure and the following cavitation. Independent of the tube size, the cavitation fronts are spreading rapidly (these move supersonically, see [27]) through the entire fluid sample. Let us assume that bacteria are subject to a number *N* of multiple, consecutive treatment stages, and that bacteria which survive any given treatment stage are completely unaffected by this; this hypothesis should be tested by further experiments and is in line with the notion that only bacteria in proximity of the tube's wall, subject to emergence and collapse of cavitation bubbles, are affected by high shear strains and therefore undergo lysis. This would imply that the final survivability rate cannot exceed *S* = s^*N*^; for example *N* = 5 would result in a survivability *S* ≈ 0.06 of the bacteria in the non-ejected fluid, *N* = 10 would result in a survivability *S* ≈ 0.003, and so on.

In the experiments presented here cavitation was initiated by the rupture of the diaphragm, however we have shown previously [27] that a relatively compliant spring-supported rigid plate mounted at the distal end of the tube would produce the same effects.

The impulses applied in this experimental study were small, of order 2 Ns, and kinetic energies were of order 250 J; such loading parameters can be achieved in engineering practice by simpler techniques than projectile impact (e.g. by electromechanical actuators). We envisage that large industrial piping systems could be sterilized by the application of compression followed by cavitation, similar to the non-ejected fluid in the experiments presented in this paper. Both electromechanical actuators and spring-supported pistons could be inserted at several locations in the piping system; the actuators could be operated synchronously and repeatedly, to rapidly generate the compression/cavitation sequence in the entire fluid volume. We shall investigate in future studies the feasibility of the envisaged technique.

## 5. Conclusion

We employed a bespoke shock tube to subject planktonic *E. coli* to two different mechanical loading sequences applied, in a single test, to separate portions of the culture. The two different loading sequences were: (i) compression followed by cavitation, and (ii) compression followed by spray. The main conclusions of the study are as follows:

— the proposed apparatus allows subjecting bacterial colonies to controllable dynamic loading pulses in a sterile laboratory environment, allowing treatment of large liquid samples at room temperature;
— the two loading sequences applied to the cells induced substantial mortality; the compression/spray loading sequence gave higher mortality on relatively small sample volumes; the compression/cavitation loading sequence gave smaller mortality but was imposed to a much larger fraction of the test sample;
— the bacteria are more sensitive to the shear strains induced by cavitation than to the volumetric strains caused by shock compression; and
— sterilization of large liquid samples and pipework can potentially be achieved at room temperature by application of shock compression followed by liquid cavitation.

## Data accessibility

All data produced is listed in table 1 of the manuscript.
